# Phosphatidylcholine from krill increases plasma choline and its metabolites in dogs

**DOI:** 10.14202/vetworld.2019.671-676

**Published:** 2019-05-16

**Authors:** Lena Burri, Knut Heggen, Andreas Berg Storsve

**Affiliations:** Aker BioMarine Antarctic AS, Lysaker, Norway

**Keywords:** choline metabolites, choline, dog, krill meal, phosphatidylcholine

## Abstract

**Background and Aim:**

Choline and its metabolites have multiple physiological roles in the body, which are important for muscle function, memory, methylation reactions, and hepatic lipid transport. This study aimed to investigate, if inclusion of phosphatidylcholine (PC) from Antarctic krill (*Euphausia superba*) can increase the concentration of choline and its metabolites in plasma of sled dogs in comparison to a control group.

**Materials and Methods:**

Ten adult Alaskan Huskies of both genders were supplemented with PC from 8% dietary krill meal inclusion for 6 weeks, while another ten dogs received no krill meal supplementation. Blood measurements of the two groups were taken at baseline and end of the study and compared for choline and its metabolite concentrations.

**Results:**

The choline concentration of the krill meal-supplemented dogs was significantly higher after 6 weeks of krill meal feeding compared to the control group (mean increase = 4.53 µmol/L in the supplemented versus 1.21 µmol/L in the control group, p=0.014). Furthermore, krill meal-supplemented dogs showed significantly more pronounced increases in betaine (p<0.001), dimethylglycine (p<0.01), trimethylamine-*N*-oxide (p<0.001), and trimethyllysine (p<0.001) compared to the control group. Significant correlations between changes in choline and changes in its metabolites were observed.

**Conclusion:**

The results showed that krill meal supplementation was associated with significantly higher plasma choline concentrations, which correlated with changed concentrations of choline metabolites.

## Introduction

**Figure-1:**
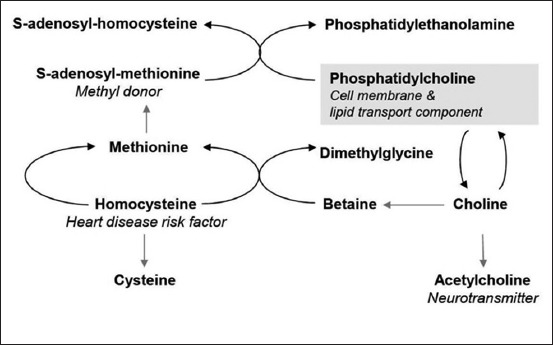
Schematic of the conversion pathways of phosphatidylcholine to its metabolites.

Choline is a conditionally essential nutrient [1]. It is needed in the synthesis of neurotransmitters (acetylcholine) for memory and control of muscle function. Likewise, choline-containing phospholipids (PLs) such as phosphatidylcholine (PC), lyso-PC, choline plasmalogen, platelet-activating factor, and sphingomyelin depend on the availability of choline. These choline-containing PLs are important for the transport of lipids, building of cell membranes, and cell membrane signaling [2]. Moreover, choline has a role in being a methyl donor. Choline is metabolized to betaine, which then provides a methyl group for the regeneration of the beneficial amino acid methionine from the heart disease risk factor, homocysteine. This reaction requires Vitamin B_12_ and folic acid as cofactors, and high homocysteine levels are linked to folate and Vitamin B deficiency [3]. Methionine is enzymatically converted to *S*-adenosylmethionine (SAMe), a universal methyl donor for various methylation reactions in the body (Figure-1). The requirement for dietary choline supplementation depends on the availability of methionine, betaine, folate, and Vitamin B_12_ due to the methyl group interrelationship. For example, betaine can replace choline addition, since choline provides the methyl group for betaine formation. Other factors that determine the dietary choline requirement are diet composition (protein, fat, and carbohydrate levels), as well as gender, age, and energy intake of dogs.

Choline metabolites control protein function and gene expression important for the proper functioning of the cardiovascular, neurological, reproductive, and detoxification systems. Many of these pathways are challenged during intense physical activity of long duration, and the demand for free, non-membrane bound choline is increased to counteract the decrease of plasma choline concentrations during endurance exercise [4,5]. Optimal muscle performance depends on sufficiently available choline for acetylcholine synthesis. It was shown that a dietary PC supplementation to increase plasma choline concentration in humans before a strenuous activity prevented the drop in circulatory choline and might improve performance [6]. The exact reasons for why choline concentrations decline during a high exercise performance are unclear but were suggested to not necessarily reflect choline utilization; instead, it might be a consequence of the redistribution of fluid pools during exertion [7].

Dogs can synthesize choline *de novo*, but this is not sufficient to meet all their bodies’ requirements, and the rest needs to be taken up from the diet in either free choline or esterified form, which reduces the need for activated methyl groups supplied by methionine.

It was found that choline in the form of PC is 12 times more efficient in raising human blood choline concentrations compared to choline salt, which is commercially available as choline chloride, choline citrate, and choline bitartrate [8,9]. While choline salt consumption in humans shows an 86% increase to a maximum plasma concentration after 30 min returning to normal levels after 4 h, PC intake raises choline by 265% and takes 12 h to decline [9].

For dogs, dietary choline is required, if the body cannot meet the demand from the synthesis of methionine, which is often the case with high-fat diets that require extensive lipid transport by PC. If an inadequate amount of choline is made and consumed, liver choline and SAMe concentrations decrease rapidly [10] and the demand for methyl groups cannot be met. In dogs, choline deficiency results in liver dysfunction due to hepatic triacylglycerol accumulation, which gets even more pronounced in diets with high-fat content [11].

A mean to increase dietary PC intake in dogs is given in krill meal that contains around 25% lipids, of which 40% are PLs, with PC being the most abundant form [12,13]. Krill are small crustaceans from the *Euphausiacea* family, which consists of 85 species [14]. Krill meal is made from the largest one, Antarctic krill (*Euphausia superba*), which is harvested in the Southern Ocean. Dietary krill meal also contains long-chain omega-3 polyunsaturated fatty acids (n-3 PUFA) and its feed inclusion has previously been shown to have the potential to improve the omega-3 index and markers for inflammation and muscle damage of Alaskan Husky sled dogs during competitive long-distance racing [15].

For the study reported here, we hypothesized that the intake of krill meal by Alaskan Huskies would increase plasma choline and its metabolites to a greater extent than would a control diet devoid of krill meal. The purpose of the study was to evaluate the potential of PC in krill meal as a dietary supplement for improving plasma choline and its metabolites in dogs.

## Materials and Methods

### Ethical approval

Guidelines by the International Animal Ethics Committee were followed, but comparing two different feeds fed to dogs, where no adverse effects of the feeds are expected, does not require approval according to the Norwegian Regulation of Animal Experimentation.

### Animals and blood sampling

A Norwegian dog sled team of 20 Alaskan Huskies took part in this study during the summer low training period, slightly increasing training in the past 3 weeks of the study. The dogs were randomly allocated into two groups, but it was ensured that there were nine males and one female in each group. One group of ten dogs received 8% of a proprietary krill dietary supplement provided by Aker BioMarine Antarctic AS (QRILL^™^ Pet, Oslo, Norway) for 6 weeks. The krill meal contained 9.6 g/100 g meal of PC+lyso-PC and the 8% inclusion into the feed increased the calculated choline concentration of the control feed from 3129 to 3262 mg/kg of diet.

Before the treatment period, the dogs were fed for 6 weeks “Labb Adult” (Felleskjøpet Agri SA, Lillestrøm, Norway), which had a choline content of 3000 mg/kg of diet. In comparison, the Association of American Feed Control Officials (AAFCO) suggests a minimum choline requirement for growth, reproduction, and maintenance of 1360 mg/kg of diet [16]. The AAFCO has not established an upper limit for choline due to lack of data for its long-term consumption and its lack of potential for overuse or toxicity. The European Pet Food Industry Federation recommends a minimum allowance for choline of 474 mg/1000 kcal ME, if the daily energy intake is 95 kcal/kg and no safe upper limit is listed [17]. The metabolizable energy of the study diets was 3768 kcal/kg, meaning that the choline content of the diets used was well above the minimum allowance of 474 mg×3.768 mg = 1786 mg choline/kg of diet. It is normal in commercial diets to include a higher content than the minimum recommended allowance to ensure a safety margin.

The second team of ten dogs received a control diet with similar n-3 PUFA content by the addition of 1.5% fish oil (NorSalmOil, Norsildmel AS, Bergen, Norway), but was devoid of krill meal. The standard feed for all dogs was based on “Appetitt Adult Maintenance” (Felleskjøpet Agri SA, Lillestrøm, Norway), a nutritionally complete, dry, and extruded diet, into which either krill meal or fish oil was added before extrusion. The two diets were formulated to be isonitrogenous and isocaloric and to meet nutrient recommendations for adult dogs (Table-1). The dogs were fed individually every evening approximately 560 g of dry food. The amount is based on experience on how much food an Alaskan Husky needs in inactive periods. They slept in separate dog houses with *ad libitum* access to drinking water.

**Table-1:** Formulation and calculated composition of experimental diets (%).

Diet	Control	Krill meal
Proteins	25	25
Krill meal^1^	-	8.21
Salmon protein silage^2^	6	6
Fish meal^3^	4	-
Chicken meal^4^	19.91	17.39
Lipids	16	16
Fatty acids		
14:0	0.30	0.32
16:0	3.15	3.20
16:1	0.62	0.61
18:0	1.03	1.02
18:1	5.00	4.97
18:2	2.59	2.56
18:3	0.26	0.25
18:4	0.04	-
20:5	0.13	0.21
22:5	0.02	0.01
22:6	0.17	0.09
EPA+DHA	3.03	3.00
∑ n-3	5.82	5.59
∑ n-6	26.31	25.96
n-6/n-3	4.52	4.64
Carbohydrates	43.68	43.80
B Vitamins		
B1	12.00	12.00
B2	24.00	24.00
B3	150.00	150.00
B5	60.00	60.00
B6	30.00	30.00
B12	0.064	0.064
Folate	4.00	4.00
kcal/kg	3764	3768

1 QRILL™ Pet, Aker BioMarine Antarctic AS, Lysaker, Norway.

2 Hordafôr AS, Bekkjarvik, Norway.

3 NorseNAT-LT, Norsildmel AS, Bergen, Norway.

4 Norsk Protein AS, Ingeberg, Norway. DHA=Docosahexaenoic acid, EPA=Eicosapentaenoic acid

Both groups underwent a physical examination by a veterinarian prior blood sample collection at baseline and end of the study about 3-4 h after feeding. During this examination, 6-8 ml of venous blood was collected from the cephalic vein into a vacutainer containing EDTA for plasma collection. Red blood cells and plasma were separated by centrifugation at 3000 rpm for 15 min at room temperature and kept on dry ice until stored at −80°C. Plasma was used for the analysis of choline and its metabolites.

The dogs participating in the study were used to regular blood sample collection due to their dog sled race experience and any discomfort was minimized by sample collection in a known environment at their kennel.

### Plasma analysis

Choline and metabolites were measured using an 1100 high-performance liquid chromatography (HPLC) system (Agilent Technologies, Santa Clara, United States of America). The HPLC system was coupled to an API3000 triple-quadrupole tandem mass spectrometer (AB Sciex, Framingham, United States of America) equipped with an electrospray ion source and fitted with a hot source-induced desolvation from IONICS (Calamba City, Philippines). Analyst (Ver.1.5.2; AB Sciex, Framingham, United States of America) was used for data acquisition and analysis. Sample processing was performed by a MicroLab AT Plus robotic workstation (Hamilton, Bonaduz, Switzerland) and samples of 10 μL of deproteinized plasma were injected onto a Fortis Phenyl column from Fortis Technologies Ltd. (Cheshire, United Kingdom) guarded by a Polar-RP SecurityGuard Cartridge (Phenomenex, Torrance, United States of America). The mass spectrometer was operated in the positive ESI mode, and analytes and internal standards were detected in multi-resolution modeling with unit resolution at quadrupole 1 (Q1) and low resolution at Q3. More details are given elsewhere [18].

### Statistical analysis

A mixed-design two-way analysis of variance (ANOVA) was employed to test for overall and interaction effects of time point (pre-supplementation at 0 weeks to post-supplementation at 6 weeks within-subjects factor) and supplement type (krill meal and control between-subjects factor) on choline levels (µmol/L). *Post hoc* independent samples t-test comparing choline change levels for the two groups across the supplementation period was subsequently carried out. Paired samples t-tests were used to test for significant changes in choline metabolites in the krill meal-supplemented group. Metabolites included homocysteine, cysteine, betaine, trimethylamine-N-oxide (TMAO), trimethyllysine (TML), dimethylglycine (DMG), methionine, creatinine, and arginine. Linear relationships between choline and its metabolites were investigated using Pearson correlations. Alpha was set at 0.05.

## Results

Twenty Alaskan Huskies with an age range from 2.5 to 5 years and an average age of 3.15 years and a mean body weight of 27.25 kg ranging between 19.1 and 32.3 kg were included in the study. Samples were compared from a group of ten dogs (nine males and one female) receiving 8% krill meal to a control group (nine males and one female) not receiving krill meal for 6 weeks. The proximate composition of dry food given to the animals is presented in Table-1. No diet-related differences were noted for food consumption or body weight over the 6-week study.

### Choline

**Figure-2:**
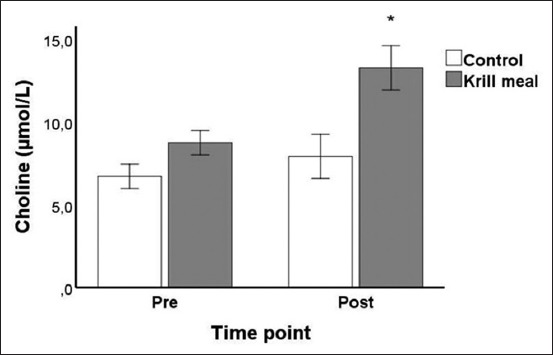
Mean (±95% confidence interval) plasma choline concentrations (µmol/L) at baseline (pre = week 0) and end of the study (post = week 6) in dogs given 8% dietary krill meal inclusion or a control diet.

A two-factor mixed-design ANOVA testing the effect of time point (pre- and post-supplementation) and supplement type (krill meal and control) on choline levels (µmol/L) revealed a significant effect of time point (F_1,18_=22.19, p<0.001) and supplement type (F_1,18_=65.08, p<0.001), as well as a significant time point × supplement type interaction effect (F_1,18_=7.44, p=0.014). Inspection of Figure-2 reveals that this interaction effect is primarily due to the krill meal group showing a much more pronounced increase in choline levels following supplementation when compared to the control group. *Post hoc* analyses confirmed this interpretation of the data: The krill meal group had a mean increase from baseline of 4.53 µmol/L versus 1.21 µmol/L in the control group, and this difference in change scores was statistically significant (p=0.014).

### Choline metabolites

Krill meal supplementation also resulted in marked changes in choline metabolites. Significant decreases across the supplementation period were found for homocysteine (p=0.017) and cysteine (p=0.045), whereas significant increases were found for arginine (p=0.023), betaine (p<0.001), creatine (p<0.001), DMG (p<0.001), TMAO (p<0.001), and TML (p<0.001). Table-2 shows a direct comparison of changes across the 6-week study period for the krill meal group and the control group, revealing that the krill meal group displayed significantly more pronounced increases in betaine (p<0.001), DMG (p<0.01), TMAO (p<0.001), and TML (p<0.001). Furthermore, an inspection of Table-3 reveals that changes in betaine, DMG, methionine, TMAO, and TML correlated significantly with changes in choline levels across both groups.

**Table-2:** Changes in choline and choline metabolites in krill meal-supplemented dogs (n=10) and control group (n=10) across the 6-week study period (μmol/L).

Plasma metabolites	Krill meal change (post-pre)	Control change (post-pre)	Difference (krill meal-control)	t (difference)	p (difference)
Choline	4.5±3.3	1.2±2.0	3.3	2.73	0.014
Arginine	31.7±36.6	61.3±33.0	−29.6	−1.90	0.073
Betaine	199.5±76.6	40.3±35.2	159.2	5.97	<0.001
Creatine	25.5±40.8	40.8±19.3	−15.3	−1.90	0.074
Cysteine	−11.8±16.1	NA	NA	NA	NA
DMG	19.2±11.2	7.7±5.6	11.5	2.90	0.009
Homocysteine	−1.7±1.9	NA	NA	NA	NA
Methionine	8.4±28.2	12.8±19.5	−4.4	0.39	0.700
TMAO	32.4±12.6	4.4±4.7	28.0	6.57	<0.001
TML	4.0±0.9	0.8±0.4	3.2	10.34	<0.001

DMG=Dimethylglycine, NA=not applicable, TMAO=Trimethylamine-N-oxide, TML=Trimethyllysine

**Table-3:** Correlations between changes (post–pre) in choline and changes in metabolites of all participating dogs (n=20).

Changes in metabolites	Changes in choline (r)	p-value
Arginine	0.41	0.07
Betaine	0.73	<0.001
Creatine	0.26	0.27
Cysteine*	0.18	0.62
DMG	0.54	0.013
Homocysteine*	0.07	0.86
Methionine	0.54	0.018
TMAO	0.70	<0.001
TML	0.54	0.013

DMG=Dimethylglycine, TMAO=Trimethylamine-N-oxide, TML=Trimethyllysine.

* =data available for krill meal group only

## Discussion

This investigation in sled dogs shows that a nutritional strategy, such as the addition of PC to the diet, can help to increase plasma choline concentrations and its metabolites. This could be of importance in a long-lasting race setting, when a drop in plasma choline is expected as seen in humans [19]. As a consequence of lowered free plasma choline concentrations, the generation of acetylcholine might be reduced and thereby negatively affect athletic performance. Noteworthy, from a performance perspective is also the significant increase in betaine in the krill group, which is thought to promote muscle function and plasma volume expansion as shown after betaine supplementation in humans in some studies, but not all [20]. However, a future study needs to investigate, if dietary krill meal inclusion can raise choline and betaine concentrations and thereby enhance physical performance during a race setting. Hence, this study can only be seen as a pilot study and has its limitations with a small number of dogs included.

Adequate choline intake is, however, not only important in high-performance dogs but also for any other dog to support body function and promote long-term health, in particular, heart, brain, and liver health. The present study has demonstrated that important choline metabolites can be significantly altered by krill meal supplementation, such as a significant reduction of plasma total homocysteine. Homocysteine is known to increase the risk of endothelial cell injury and cardiovascular disease in humans by increasing reactive oxygen species and altering lipoprotein metabolism [21], and a similar correlation has been identified in dogs [22]. Another metabolic derivative of choline that was significantly increased in the krill meal-supplemented dogs was DMG, which was suggested to have possible protective effects on glucose metabolism [23]. The potential of dietary krill meal intervention to regulate plasma DMG concentrations in dogs in the management of glycemia and insulin resistance deserves attention in future studies. Furthermore, a more direct comparison of equal amounts of choline from PC versus choline salt should be addressed in a next study since it was suggested that 60% of choline in inorganic salts is lost to conversion to trimethylamine (TMA) by intestinal bacteria, which is mostly excreted in the urine [24]. On the other hand, choline in the form of PC is considerably less converted to TMA [25], potentially making it a better choline delivery molecule.

## Conclusion

Six weeks of krill meal supplementation in dry, extruded food to sled dogs significantly increased plasma concentrations of choline, betaine, DMG, methionine, and TMAO and decreased homocysteine. How much of the TMAO was made from choline by gut microbiota is, however, unclear, since it is also a component of krill meal.

The results of this study are encouraging for krill meal supplementation not only of dogs that perform long-distance races, but also of dogs that have health ailments affecting the brain, heart, and liver. However, the exact impact of krill PC supplementation on endurance and health issues of dogs and its comparison to choline salts need to be confirmed in more focused studies.

## Authors’ Contributions

KH and LB designed the study protocol and were involved with sample collection, LB wrote the paper, and ABS performed the data analysis and was involved with drafting of the manuscript. All authors revised, read, and approved the final manuscript.
